# Structural features based genome-wide characterization and prediction of nucleosome organization

**DOI:** 10.1186/1471-2105-13-49

**Published:** 2012-03-26

**Authors:** Yanglan Gan, Jihong Guan, Shuigeng Zhou, Weixiong Zhang

**Affiliations:** 1 Department of Computer Science and Technology, Tongji University, Shanghai, China; 2 Shanghai Key Lab of Intelligent Information Processing and School of Computer Science, Fudan University, Shanghai, China; 3 Department of Computer Science, Washington University in St. Louis, St. Louis, USA; 4 Department of Genetics, Washington University School of Medicine, St. Louis, USA

## Abstract

**Background:**

Nucleosome distribution along chromatin dictates genomic DNA accessibility and thus profoundly influences gene expression. However, the underlying mechanism of nucleosome formation remains elusive. Here, taking a structural perspective, we systematically explored nucleosome formation potential of genomic sequences and the effect on chromatin organization and gene expression in *S. cerevisiae*.

**Results:**

We analyzed twelve structural features related to flexibility, curvature and energy of DNA sequences. The results showed that some structural features such as DNA denaturation, DNA-bending stiffness, Stacking energy, Z-DNA, Propeller twist and free energy, were highly correlated with in vitro and in vivo nucleosome occupancy. Specifically, they can be classified into two classes, one positively and the other negatively correlated with nucleosome occupancy. These two kinds of structural features facilitated nucleosome binding in centromere regions and repressed nucleosome formation in the promoter regions of protein-coding genes to mediate transcriptional regulation. Based on these analyses, we integrated all twelve structural features in a model to predict more accurately nucleosome occupancy in vivo than the existing methods that mainly depend on sequence compositional features. Furthermore, we developed a novel approach, named DLaNe, that located nucleosomes by detecting peaks of structural profiles, and built a meta predictor to integrate information from different structural features. As a comparison, we also constructed a hidden Markov model (HMM) to locate nucleosomes based on the profiles of these structural features. The result showed that the meta DLaNe and HMM-based method performed better than the existing methods, demonstrating the power of these structural features in predicting nucleosome positions.

**Conclusions:**

Our analysis revealed that DNA structures significantly contribute to nucleosome organization and influence chromatin structure and gene expression regulation. The results indicated that our proposed methods are effective in predicting nucleosome occupancy and positions and that these structural features are highly predictive of nucleosome organization.

The implementation of our DLaNe method based on structural features is available online.

## Background

In an eukaryotic nucleus, chromosomes are organized in condensed chromatin structures. The genomic DNA sequence wraps on a histone octamer to form primary repeating units of chromatin, termed nucleosomes. In many species, each nucleosome core particle consists of roughly 147 base pairs [1], which facilitates the storage and organization of long eukaryotic chromosomes. Nucleosome distribution on genomic DNA sequences can greatly affect gene transcription, DNA replication and reparation, by modulating the accessibility of underlying DNA sequences to various regulatory factors [2]. However, how nucleosome organization is established has not been well understood.

Besides a multitude of factors, including chromatin remodelers [3-5] and specific DNA-binding proteins [6,7], intrinsic DNA sequence preferences have been the focus of recent experimental and bioinformatical studies, which concern how and to what extent sequence features contribute to nucleosome organization [8-14]. In particular, AT- and GC-riched dimeric and trimeric motifs were first identified by the pioneer work of Trifonov [15]. Subsequently, several studies delineated periodicity and sequence patterns associated with nucleosomal sequences [8,10,11]. Specifically, G + C content can explain ~50% of the variation of nucleosome occupancy in vitro [10]. Computational methods based on such sequence compositional features have been proposed to predict nucleosome occupancy [8,9,12-14]. However, it has been demonstrated that DNA sequence preferences for certain sequence motifs are not the major determinants of nucleosome organization [16,17], which raise a question about the role of the structural variability of DNA sequences in the formation of nucleosomes [10,18-20].

To address this question, several studies have been geared toward structural properties of DNA sequences and the conformation mechanism of nucleosomes. Some physicochemical properties in nucleosomal DNA databases, such as tilt for DNA-protein complex and helical twist, have been identified to be significant for nucleosome binding [21]. Based on the roll-and-slide model, Tolstorukov et al. found that slide of adjacent base pairs contributed predominately to DNA super-helical pitch and roll of neighboring base pairs accounts for DNA curvature [22]. Miele et al. introduced dinucleotide-dependent DNA flexibility and intrinsic curvature to the analysis of nucleosome occupancy [20]. Morozov et al. used a DNA elastic energy function to build a biophysical model of sequence dependence of nucleosome formation [23]. The bendability of dinucleotides in the crystal structures of DNA duplexes was also analyzed within nucleosomal DNA fragments [24,25]. Analysis of nucleosome crystal structures showed that the behaviors of base pairs, puckering of ribose rings and related backbone torsion jointly represent the major structural variations of nucleosomal DNA sequences [26]. These studies suggested that there might exist many structural features related to nucleosome formation. Therefore, it is imperative to systematically analyze different structural properties and identify structural features that contribute to nucleosome formation and more importantly, to understand to what extent nucleosome organization is inherently hardwired in these structures of genome sequences. Furthermore, it is desirable to exploit those structural features that are characteristic of nucleosome occupancy and formation to develop effective novel methods for predicting nucleosome positioning.

We systematically investigated twelve structural features related to intrinsic flexibility, curvature and energy of DNA sequence, and analyzed their relation with nucleosome occupancy, chromatin organization and transcriptional regulation across the entire *S. cerevisiae *genome. By focusing on centromere and promoter regions, we further inquired into the underlying structural mechanisms of nucleosome organization and transcriptional regulation. To assess their predictive power for nucleosome organization, we combined these structural features in a linear model for predicting nucleosome occupancy. Further, we introduced a novel strategy to locate nucleosomes by detecting peaks of structural profiles, and developed a meta predictor to integrate information from different structural features, which significantly outperformed the existing sequence-based methods. We also constructed an alternative, hidden Markov model (HMM) for predicting nucleosome positions using the structural features, confirming the effectiveness of these structural features in locating nucleosomes. Our results shed lights on the recent debate on the role of sequence preference in nucleosome organization [9,27,28], indicating that DNA structures are important factors for determining nucleosome organization.

## Results

### Structural features correlate with global nucleosome occupancy

To decipher the code of intrinsic chromatin organization from a structural perspective, we examined a dozen thermo-physical features of DNA sequences, listed in Table 1. According to different structure models derived from biochemical experiments, these features characterize various structural aspects of DNA sequences, including flexibility, curvature and folding energy. In particular, the propeller twist angle scale is calculated by X-ray crystallography of 60 kinds of different DNA oligomers, to capture the conformational flexibility of dinucleotides [29]. The B-DNA twist measures the mean twist angles in B-DNA [30]. As enzyme Dnase I is inclined to bind to the minor groove and to cut DNA that is bent, Dnase I cutting frequencies measure the bendability of DNA sequences [31]. Protein-induced deformation reflects the deformability of the DNA helix changed by proteins [32]. Protein-DNA twist describes the DNA variability [32]. The DNA-bending stiffness is regarded as the translational positioning of nucleosomes [33]. The model of base-stacking energy is derived from approximate quantum mechanical calculations on crystal structures, measuring dinucleotide base-stacking energy [34]. DNA denaturation is quantified by the melting temperature of helix denaturation [35]. A-philicity represents the free energy required for transition from B- to A-DNA conformation [36], and Z-DNA is related to the free energy required for transition from B- to Z-DNA transitions [37]. Duplex disrupt energy reflects the stability of a DNA duplex [38]. Duplex free energy is calculated as the transition enthalpy of the melting behavior of different duplex [39]. Although these structural models are based on dinucleotide or trinucleotide parameters, several studies have proven that these structural features are in fact not exactly the same as the nucleotide information and offer additional thermo-physical information [40-42]. These structural features capture long-range interactions which are beyond short local sequence features [43], and are complementary to each other [40]. Typically, these features have been shown to be effective in promoter prediction and have revealed differences in information content of delineating promoter regions [42,44]. Here we studied these structural features and their impact on chromatin organization in model species *S. cerevisiae*.

**Table 1:** Twelve structural features of DNA sequences, and genome-wide correlation coefficients between in vitro and in vivo experimental nucleosome occupancies [9,45] and structural profiles of 12 features

Structural features	Description	Pearson correlation		
		
		**In vitro **[9]	**In vivo **[9]	**In vivo **[45]
Propeller twist [29]	The angle of the two aromatic bases in a base pair.	0.82	0.67	0.35

DNA denaturation [35]	The ability of DNA to denature.	0.77	0.61	0.34

DNA-bending stiffness [33]	The anisotropic flexibility of DNA.	0.72	0.56	0.35

Bendability [31]	The trinucleotide bendability.	0.63	0.51	0.15

Duplex disrupt energy [38]	DNA duplex energy.	0.57	0.40	0.21

Stacking energy [34]	Energy scale of dinucleotide base-stacking energy scale.	-0.80	-0.63	-0.35

Z-DNA [37]	The ability to be covered from B-to Z-DNA	-0.78	-0.61	-0.36

Duplex free energy [39]	The thermodynamic energy content.	-0.74	-0.57	-0.33

Aphilicity [36]	The free energy values for a transition from B- to A-DNA form.	-0.69	-0.54	-0.27

Protein-DNA twist [32]	The ability to be deformed by protein.	-0.52	-0.42	-0.16

B-DNA twist [30]	The mean twist angles in B-DNA.	-0.17	-0.11	-0.08

Protein-induced deformation [32]	The ability to be changed by proteins.	-0.09	-0.06	-0.02

These structural features are classified into two classes, positively correlated (upper part) and negatively correlated features (lower part)

First, we computed and compared the structural profiles of all these 12 structural features on 1,000 well-positioned nucleosomes and 1,000 nucleosome-depleted sequences [45] (see Methods). The results show that nucleosome-enriched sequences have different structural characteristics from that of nucleosome-depleted sequences. Based on their relationship with nucleosome occupancy, we can classify these structural features into two categories. As shown in Table 1 the first class of features are positively correlated with nucleosome occupancy. For each feature in this class, the calculated structural values along nucleosome sequences are greater than that along nucleosome-depleted sequences. In contrast, the structural features in the second class show negative correlations with nucleosome occupancy. Take DNA denaturation as an example, this feature captures the temperature at which DNA strands are half denatured and DNA regions with a low denaturation value denaturate more easily than regions with a higher value [35,44]. Therefore, this feature can measure the stability of a double DNA strand. The results reveal that nucleosome-enriched DNA sequences denature at a higher temperature than nucleosome-depleted DNA sequences. In contrast, we observe that the duplex free energy of nucleosome sequences is evidently lower than that of nucleosome-depleted sequences. It is well known that DNA sequences with a low free energy is more stable than that with a high free energy [39,44]. That is to say, a DNA segment in nucleosome is more stable than nucleosome-depleted sequences [20].

**Figure 1:**
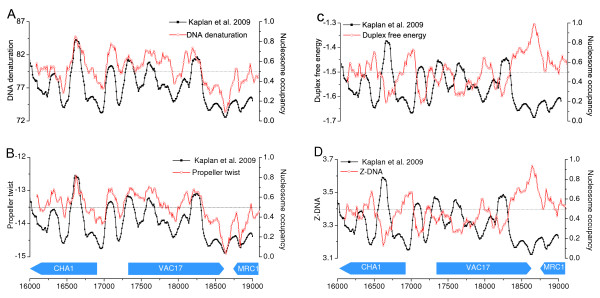
**Calculated structural profiles (red line) and in vitro experimental nucleosome occupancy (black line) in a 3 kb region around CHA1 promoter of *S. cerevisiae *(Chr. 3)**. Shown are two positively correlated features (A) DNA denaturation and (B) Propeller twist, and two negatively correlated features (C) Duplex free energy and (D) Z-DNA.

Furthermore, we directly compared the calculated profiles of the individual structural features and in vitro experimental nucleosome occupancy data [9] along the whole genome of *S. cerevisiae*. Here, we plot the results around two benchmark loci. Figure 1 shows four representative features on chromosome 3 around CHA1 promoter. In the figure, the values for the experimental data represent the nucleosome coverage along the sequence; a peak represents the position where a nucleosome is potentially located, while a valley region corresponds to a nucleosome-depleted sequence. The results of the other features and the results on another well characterized region surrounding HIS3 promoter on chromosome 15 are respectively included in Additional file 1: Figures S1 and S2.

We observe in Figures 1(A) and 1(B) that the peaks and valleys for the positively related DNA denaturation and Propeller twist align well with the experimental nucleosome signals, both of which share very similar patterns with experimentally determined nucleosome occupancy. Figures 1(C) and 1(D) compare the profiles of negatively related structural features with the experimental nucleosome occupancy. As shown, the patterns of the actual nucleosome occupancy and the profiles of structural features are rather opposite. Specifically, the local valleys of the structural profiles correspond well to the peaks of experimental nucleosome signals. As a support to the above finding, the plot shows that nucleosome-enriched sequences indeed have different structural patterns from nucleosome-depleted sequences. In eukaryotic cells, promoter regions are normally less likely to be occupied by nucleosome, making them more accessible to the transcription machinery [46,47]. The structural profiles we computed agree very well with this finding. For positively related features, deep valleys are located in the promoter regions, while peaks are observed for negatively related features. Taken together, these comparative results show that these structural patterns correlate to different degrees with the experimental nucleosome occupancy.

To quantify the power of structural features for capturing nucleosome occupancy signals, we analyzed the correlation between the structural profile of each feature and experimental nucleosome occupancy along the whole genome of *S. cerevisiae*. Specifically, we collected one in vitro [9] and two in vivo [9,45] genome-wide nucleosome occupancy datasets as reference. The Pearson correlation coefficients, listed in Table 1 confirmed the results of our classification of the structural features that we studied. The result on nucleosome formation energy agreeed with the previous results from different models [20,23], showing that nucleosome-energy is highly correlated with nucleosome occupancy. Furthermore, we analyzed other structural features related to DNA flexibility and intrinsic curvature in order to identify the features that contribute the most to nucleosome formation. Among the structural features we studied, Propeller twist, DNA denaturation and DNA-bending stiffness are the most positively correlated with nucleosome occupancy, and Stacking energy, Z-DNA and Duplex free energy are the most negatively correlated features. The close correlations between these structural features and nucleosome occupancy imply that these features are important factors of in vitro and in vivo nucleosome organization. Meanwhile, unlike in vitro situation, in vivo nucleosome occupancy data is less correlated with the structural features, suggesting that nucleosome organization may also be influenced by the action of additional external factors like DNA binding proteins and chromatin remodelers [48].

Since these features capture different aspects of nucleosome occupancy, we thus examined to what extent these features are correlated with each other. We calculated the pairwise Pearson correlation coefficients of these 12 features. The results, presented in Additional file 1: Table S1, show that there are close correlations among DNA denaturation, DNA-bending stiffness and energy-related features. Features measuring energy (Duplex free energy, Duplex disrupt energy, Stacking energy and Stabilizing energy of Z-DNA) are highly correlated with each other. Propeller twist, Aphilicity and other features are less correlated. These results demonstrate that these twelve features capture different structural dimensions of DNA sequence and have unequal capability for capturing nucleosome occupancy.

Previous analyses have shown that the G + C content is one of the most important features, which can explain approximately 50% the variation of in vitro nucleosome occupancy [10]. To understand whether the effectiveness of these structural features that we studied depends on the G + C content, we studied the correlation of these structural features with the G + C content on the whole genome and in promoter and genic regions. As shown in Additional file 1: Table S2, the G + C content is correlated with some of the structural features, such as Aphilicity, Bendability, DNA-bending stiffness and the energy-related features. However, the corresponding Pearson correlation coefficients are not proportional to their performance of predicting nucleosome occupancy and positions. For example, although the Bendability and Duplex disrupt energy are highly correlated with the G + C content, they are not effective in capturing nucleosome occupancy (Table 1). Meanwhile, the correlation in the nucleosomedepleted promoter regions is higher than that in the nucleosome-enriched gene regions. All these results indicate that the effectiveness of these structural features is just marginally related to the G + C content, suggesting that the G + C content may be less important than we have thought [49] and some of the structural features may capture information of nucleosome occupancy beyond the G + C content.

### Structural features and nucleosome occupancy in centromere region

To analyze whether intrinsic encoding of nucleosome occupancy varies across different types of chromosomal regions, we next focused on two representative kinds of local genomic regions, nucleosome-enriched centromere region and nucleosome-depleted promoter region. The centromere of a eukaryotic chromosome, which accommodates sites for segregation during mitosis and meiosis, is one of the essential parts of chromosome. Previous research revealed that a centromere region has high nucleosome occupancy [8]. A key question is what determines the nucleosome occupancy over centromere regions.

**Figure 2:**
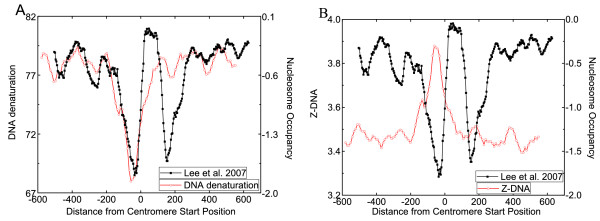
**Computed average structural features (red line) and average in vivo experimental nucleosome occupancy (black line) of all centromere regions in *S. cerevisiae *genome**. Shown are the results on one positively correlated feature (A: DNA denaturation) and one negatively correlated feature (B: Z-DNA).

We analyzed all centromere regions in the *S. cerevisiae *genome. Unlike the centromeres of other eukaryotes with long and highly repetitive DNA sequence, *S. cerevisiae *centromeres are short (about 120 bp). However, they still possess distinct signatures. Based on the experimentally determined nucleosome locations [45], we found that there exists a stable nucleosome around each of the centromere regions, and the average offset between a nucleosome and a centromere is less than 20 bp. We further analyzed the structural features around centromere regions. In order to find some common structures of centromere regions, we calculated the average structural profiles of all centromere regions in the *S. cerevisiae *genome. Figure 2 shows two representative structural features and experimental nucleosome occupancy data around the centromere region [45]. As shown in Figure 2(A), the DNA denaturation value over the centromere is higher than that over other region, implying that the DNA sequence denatures harder at the centromere. For features that negatively correlate with nucleosomes, there exist evident valleys over the centromere regions (Figure 2(B)). Overall, the structural profiles are highly correlated with nucleosome occupancy around the centromere regions. These structural features to some extent dictate the high nucleosome occupancy over centromere region and enhance the stability of histone-DNA interactions [8].

### Structural features and nucleosome occupancy in promoter regions and the effect on gene expression

Our analysis indicated that different genomic regions have distinct structural properties, which may dictate nucleosome occupancy patterns specific to these regions. Specifically, the regions upstream of transcription start sites (TSS) have less DNA-bending stiffness and Propeller twist, which may lead to more depletion of nucleosome than the corresponding downstream regions. Several independent studies further revealed that nucleosome depletion in promoter regions was related to gene regulation [2,19,45]. Given the correlation between nucleosome occupancy and the structural features we studied, variability in gene expression might be inherently hardwired in structural properties of promoters. To investigate whether genes with a similar expression pattern share some chromatin structures, we categorized genes on the basis of their expression levels and calculated the average structural profiles of promoter regions of 5,015 high-confidence transcripts of *S. cerevisiae *reported in [45,50].

**Figure 3:**
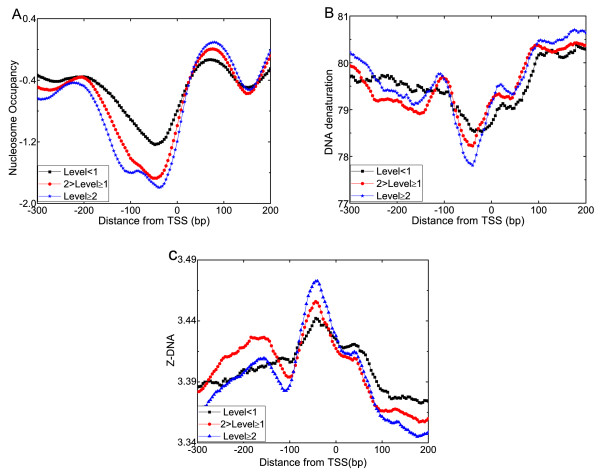
**Variation of gene expression correlates with local in vivo nucleosome occupancy and DNA structures**. Plotted are the results in the 500 bp region surrounding transcription start sites (TSS) of 5,015 differentially expressed genes. The 5,015 genes were grouped into 3 classes by their expression level (EL): EL *<*1 (n = 759, black line), 1 ≤ EL *<*2 (n = 1,859, red line) and EL *≥ *2 (n = 2,397, blue line). (A) Average in vivo nucleosome occupancy. (B) Average structural profiles of DNA denaturation. (C) Average structural profiles of Z-DNA.

Based on the experimentally determined nucleosome data [45], we computed average nucleosome occupancy with respect to different gene expression level. The result in Figure 3(A) shows that the patterns of nucleosome occupancy within upstream regions and downstream regions of TSSs are antagonistic. The gene expression level is negatively correlated with the degree of nucleosome occupancy at -1 nucleosome positions, but is positively correlated with that at +1 nucleosome positions. A possible explanation is that the promoters of transcribed genes need ordered nucleosome structures within the coding regions, which perhaps increase residence time of the Rpd3S complex [45]. Furthermore, we analyzed the underlying relationship from a structural viewpoint. For structural features positively correlated with nucleosome occupancy, the structural values in regions upstream of TSSs are negatively correlated with the gene expression levels. The plot in Figure 3(B) indicates that highly expressed genes maintain a low DNA denaturation ability in the critical promoter regions, compared with the higher stability of less active genes. In contrast, the structural profiles of negatively correlated features are completely opposite. As shown in Figure 3(C), highly expressed genes preferentially have the Z-DNA structure in the regions upstream of TSSs. As we used a sliding window (100 bp) to smooth out the structural values, there was a smoothing effect for the structural profile. For the DNA denaturation, the smoothing effect produced a small valley around +50 bp and a small peak at +20 bp downstream of the big valley at -1 nucleosome position. For the Z-DNA structure, the big peak (at -1 nucleosome position) of the structural profile led to a small peak at +50 bp downstream of TSS, which was also resulted from the smoothing effect. However, the overall pattern of the structural profiles within promoter regions was closely associated with gene expression activities. These observations are in line with the view that promoters of expressed genes possess specific structures, presumably to occlude nucleosome formation and permit transcription factors binding. These findings imply the potential of predicting nucleosome binding events and expression patterns from secondary structures of DNA sequence.

### Structural features are highly predictive of nucleosome occupancy

Intrigued by the high degrees of correlation of the 12 structure features with the experimental nucleosome occupancy, we adopted the least angle regression method (abbreviated as LARS) [51] to combine the structural features in a linear model for predicting nucleosome formation potential. The LARS algorithm determines a linear combination of the structural features by optimizing a linear model with a set of training data. In the model, the coefficients of the features specify which features are used and their relative weights in the combination, and the output gives rise to the prediction to nucleosome occupancy. Then we generated a structural feature-based nucleosome occupancy prediction model. In our implementation, we used the version of LARS in the R package [52]. Particularly, we trained three linear models on chromosomes 1-9 using one in vitro dataset [9] and two in vivo datasets [9,45] of nucleosome occupancy dataset, and applied the resulting models to predict nucleosome occupancy on chromosomes 10-16. The predicted nucleosome occupancy and the in vitro data are highly correlated, with a Pearson correlation coefficient of 0.88. For the in vivo nucleosome occupancy, the correlations are respectively 0.75 and 0.42 on Kaplan et al's dataset and Lee et al's dataset. The result shows the models based on these structural features are highly predictive of in vivo and in vitro nucleosome occupancy. However, the performance of these structural features for predicting in vivo nucleosome occupancy is not as good as for the in vitro nucleosome occupancy. This result indicates that in vivo nucleosome organization may also be influenced by other factors such as DNA methylation, histone variants, chromatin remodelers and DNA-binding proteins [53].

To further evaluate the performance of our new integrated models, we compared them with eight recent published prediction models [2,9,10,14,20,25,45,54], part of which were also used in Tillo and Hughes's study [10]. As described in Table 2 most of the previous models depended on sequence compositional information, such as k-mers preference, periodic dinucleotides [2,9,14,54]. Tillo et al's model and Lee et al's model both combined many kinds of features, such as G + C content, 4-mers occurrence and a few structural features. Miele et al's model computed the sequence-dependant free energy of nucleosome formation based on DNA flexibility and intrinsic curvature [20]. Gabdank et al's model utilized the DNA bendability matrix to map nucleosomes on genomic sequences [25]. Different from the previous works, our model focused on systematically analyzing the effectiveness of twelve kinds of structural features in capturing nucleosome occupancy. Since the models that we compared were not developed using the same set of data, it is difficult to choose a benchmark dataset for evaluating their performance. In order to compare with the previous results, we used the same in vitro [9] and in vivo [45] datasets as in Tillo and Hughs's study [10]. The comparison results are summarized in Table 2 showing that the performance of our integrated model for in vitro nucleosome occupancy is comparable with the models devised by Kaplan et al [9] and Tillo et al [10]. For in vivo nucleosome occupancy prediction, our model outperformed the other existing models, except Lee et al's model. Besides structural features, Lee et al's model also included G + C content, 4-mers occurrence, and TFBSs, which may lead to a slight better performance than our model in vivo. However, our model significantly outperformed Lee et al's model in predicting in vitro nucleosome occupancy. In addition to free energy and Propeller twist, which were used in the previous studies, our models also assigned high weights to DNA denaturation, DNA-bending stiffness, Stacking energy and Z-DNA, indicating that they are effective in capturing nucleosome occupancy. Specifically, the performance of our model is better than that of the previous models based on the energy or bendability. The difference of performance may attribute to two factors. On one hand, the structural features used in these methods are calculated by different structural model. On the other hand, our combination model of the structural features is effective. These results reveal the importance of that these structural features in capturing nucleosome occupancy. According to their mutual correlations, the linear model combining these complementary features can capture different structural dimensions of DNA sequences, which may contribute to the prediction of nucleosome occupancy.

**Table 2:** Genome-wide correlation coefficients between experimental nucleosome occupancies and nucleosome occupancies predicted by different models

Prediction models	Features used in a model	Pearson correlation	
		
		**In vitro **[9]	**In vivo **[45]
Our integrated model (this paper)	12 structural features in a linear model	0.88	0.42

Xi et al., 2010 [54]	Position-dependant k-mer preferences (k up to 5)	0.618	0.34

Kaplan et al., 2009 [9]	Position-dependant 5-mer preferences and periodic dinucleotide	0.89	0.34

Tillo and Hughes, 2009 [10]	A linear model combining G + C content, propeller twist, slide and several 4-mer occurrence	0.86	0.38

Yuan and Liu, 2008 [14]	Periodic dinucleotide signals of linker and nucleosomal sequence	0.35	0.27

Gabdank et al.,2010 [24]	Uses DNA bendability matrix	0.41	0.25

Miele et al., 2008 [20]	Sequence-dependant free energy of nucleosome formation	0.38	0.22

Field et al., 2008 [2]	Uses 5-mer preferences and periodic dinucleotide	0.74	0.39

Lee et al., 2007 [45]	G + C content, 4-mer occurrence, TFBSs and several structural features	0.63	0.42

### Structural features are highly predictive of nucleosome positions

So far we have observed that the profiles of structural features we analyzed are well correlated with experimental nucleosome occupancy data. Take the propeller twist feature as an example, most nucleosome regions have a peak in this profile and there is virtually no peak in nucleosome-depleted regions. This indicates that the structural properties are sufficiently distinct to allow efficient prediction of nucleosome positions. We thus developed a computational method, termed DLaNe, for detecting peaks and valleys of structural profiles to locate nucleosome positions. Specifically, for positively correlated features, our method detects peaks along the structural profiles to locate nucleosome; likewise, it detects valleys for negatively related features. Meanwhile, as nucleosome positions are influenced not only by high order chromatin structure [53], but also by repulsive and attractive interactions between neighboring nucleosomes [55], we considered the effect of the steric exclusion which prevents neighboring nucleosomes from overlapping in space [8] and dictates relatively fixed lengths of linker DNA. In yeast, the average length of nucleosome is about 147 bp, and the length of linker DNA ranges approximately in 10-20 bp [56]. We set the window size for nucleosome position prediction at 165 bp to count for the distances between neighboring nucleosomes. In our analysis, we experimented with different window sizes. The results showed that this particular window width performed the best. The detail of our method is in Methods.

We applied our method to the *S. cerevisiae *genome. To determine the predictive power of different structural features, we validated our predicted nucleosome locations against the genome-wide nucleosome position map from Lee et al. [45], which provided 70,884 nucleosome positions at a 4 bp resolution from a tiling microarray. If a predicted nucleosome center is within *L *bp of a true site, we took it as a correct prediction, where *L *is a parameter of distance cutoff. To obtain a fair evaluation, we evaluated predicted positions by different distance cutoffs. We used six cutoff values, ranging from 10 bp to 60 bp with an increment of 10 bp. As previous studies evaluated their prediction accuracy in terms of sensitivity and specificity [13,57], here we also adopted these criteria. Specifically, sensitivity (*Se*) represents the fraction of experimentally verified nucleosomes that are correctly predicted, and specificity (*Sp *) is the fraction of correctly predicted nucleosomes out of all predictions. In addition, to compare the performance of methods with different *Se *and *Sp*, a unified *F-measure *was used, computed as 2·*Se*·*Sp*/(*Se *+ *Sp*).

**Figure 4:**
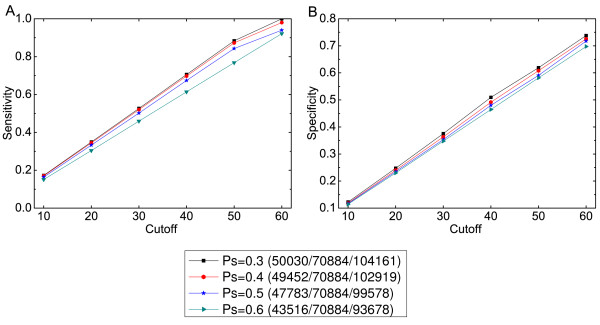
**Performance of our method DLaNe with different peak significance *(Ps) *values**. Here we present the results of four thresholds at six different distance cutoff values from 10 to 60. (A) Sensitivity. (B) Specificity.

Since our method depends on a peak significance threshold to identify peaks (see Method), whose center is further used to determine nucleosome position, we firstly examined whether the performance was influenced by this parameter. A larger threshold means a more stringent standard and more significant peaks to be detected. Figure 4 shows the results for different thresholds. To clearly show the effect of each threshold, we correspondingly list the number of correctly predicted nucleosomes, the number of reference nucleosomes [45] and the total number of predicted nucleosomes in the legend. We present these three counts at the cutoff *L *= 40 bp, and the results for the other cutoff values are similar (not shown). From these plots, we observe that both the sensitivity and specificity decrease slightly as the threshold increases. The reason is that increasing the threshold can filter out more peaks and thus lower the total number of prediction.

We then tested how well these structural features predict concrete positions of nucleosomes along the *S. cerevisiae *genome. Each feature was individually utilized to construct a DLaNe model. Table 3 reports the prediction accuracies of all of the 12 structural features. The experimental results reveal that the structural features we considered can indeed be exploited to predict nucleosome positions. Regardless of whether positively or negatively correlated, the predictive accuracies of different features are generally consistent with their correlations with nucleosome occupancy. DNA denaturation, Propeller twist, Stacking energy and Z-DNA have the highest performance among all features, while Protein deformation and Protein-DNA twist have the lowest performance.

**Table 3:** Genome-wide performance comparison among the Segal method, N-score, NuPoP, the Random method, the HMM method, DLaNe based on twelve individual structural features and the meta DLaNe method combing six features with the cutoff *L *= 35

Structural features	Prediction performance			
	
	Se	Sp	F-measure	Improvement(%)
DNA denaturation	0.703	0.412	0.520	18.47

Propeller twist	0.699	0.409	0.516	17.68

DNA-bending stiffness	0.702	0.408	0.516	17.68

Duplex disrupt energy	0.625	0.394	0.483	10.21

Bendability	0.623	0.391	0.480	9.56

Z-DNA	0.702	0.411	0.518	18.23

Stacking energy	0.695	0.408	0.514	17.25

Duplex free energy	0.689	0.404	0.509	16.15

Aphilicity	0.675	0.403	0.505	15.09

B-DNA twist	0.654	0.384	0.484	10.34

Protein-DNA twist	0.652	0.381	0.481	9.67

Protein deformation	0.526	0.353	0.422	-3.66

Meta DLaNe method	0.734	0.457	0.563	28.45

HMM method	0.723	0.445	0.551	25.63

Segal method	0.474	0.408	0.439	0.00

NuPoP method	0.356	0.489	0.412	-6.04

N-score method	0.317	0.439	0.368	-16.05

Random method	0.346	0.346	0.346	-21.10

The accuracy of a predicted nucleosome position is measured by sensitivity (*Se*), specificity (*Sp*) and F-measure. The improvement of F-measure is computed by comparing with the performance of the pioneer method (Segal method). For DLaNe method, the performance of all 12 structural features is shown in two part, according to their relationship with nucleosome occupancy

**Figure 5:**
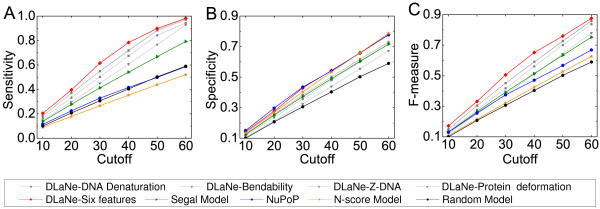
**Performance comparisons of the new method DLaNe with three recently methods and the random method with different distance cutoffs**. (A) Sensitivity. (B) Specificity. (C) F-measure.

Further, we evaluated the performance of our DLaNe method by comparing it with three recent computational nucleosome prediction methods [8,14,54] and a random model as a reference point. The Segal method used a position-dependant first-order Markov chain to locate nucleosomes [8]. The N-score method utilized wavelet energy to identify a multi-resolution sequence signature, and then applied a hidden Markov model (HMM) to predict nucleosome locations [14]. We obtained all highly stable nucleosome positions (stability scores larger than 0.2) predicted by the Segal method and predictions of N-score. The NuPoP method was built upon a duration HMM [54]. As suggested, we ran NuPoP with its fourth order Markov model to predict nucleosome positions [54]. For the random model, we randomly selected the same number of non-overlapping nucleosome positions from each chromosome as in the reference map [45]. All nucleosome positions predicted by different methods were equally validated by the genome-wide reference nucleosome positions [45]. Limited by space, we unbiasedly included the best and the worst predictive structural features in this comparison. Performances under different distance cutoffs show that our meta DLaNe achieves the highest sensitivity and F-measure (Figure 5). Table 3 shows the detailed comparison with cutoff L = 35 bp. For the most informative features such as Z-DNA and DNA denaturation, the number of correct predictions from DLaNe is almost twice of that of N-score, and about 25% more than that of the Segal method. The F-measure of DLaNe, except the feature Protein deformation, is always higher than that of other methods. Specially, the F-measure of the meta DLaNe, which combines six top-performing features, is 28.45% higher than that of the Segal model, 36.71% higher than that of NuPoP, about 50% higher than that of N-score and Random method. These comparisons reveal that DLaNe method is very effective in locating nucleosome positions.

To determine the factors that make the meta DLaNe perform better than other methods, we also applied the HMM approach to locate nucleosomes using the structural profiles of the six top informative features (see Methods). The HMM model contained 16 hidden states: 15 nucleosome states and one linker state. We trained the model on Chromosome 3 and applied it to predict nucleosome positions by using Viterbi algorithm. As shown in Table 3 the HMM model performs slightly worse than the meta DLaNe, however, better than the existing method which mainly based on sequence features. Since this HMM method and the DLaNe are both based on structural features, the results suggest that these structural features are effective in capturing nucleosome positioning information.

## Discussion

It has been heatedly debated whether or not nucleosome organization is primarily determined by genomic DNA sequences [8,27,28]. By analyzing nucleosome occupancy in yeast, Kaplan et al concluded that DNA sequence preferences have a dominant role in nucleosome organization [9,27]. However, subsequent studies derived a different conclusion [16,17,28]. The main dispute is to what extent sequence preferences dictate nucleosome organization. In the current study, we systematically investigated 12 structural properties of DNA sequences, including flexibility, curvature and energy, as features for nucleosome occupancy. We have identified some critically important structural features, such as DNA denaturation, DNA-bending stiffness, Stacking energy, Z-DNA, Propeller twist and free energy, which are not only highly correlated with in vitro nucleosome organization, but also accounted for much of the in vivo nucleosome occupancy. The correlation analysis between the 12 structural features and the G + C content of DNA sequences showed that the predictive power of these structural features just marginally related to the G + C content. Besides sequence compositional preferences, such as the G + C content, these structural features can also capture long range interactions that are invisible in local sequences.

Our study provided some new structure-based perspectives on nucleosome organization and gene regulation activities. Firstly, the genome-wide profiles of these 12 structural features are highly correlated with both in vitro and in vivo nucleosome occupancy. Based on their relation with nucleosome occupancy, these features are classified into two categories, positively and negatively correlated. The peaks of structural profiles for positively correlated features well correspond to nucleosome regions and the valleys match nucleosome-depleted ones, while negatively correlated features are the opposite. This suggests that structural properties of DNA sequence would directly determine nucleosome occupancy. These structural features differ in degrees of correlation with nucleosome occupancy. Secondly, the analysis over centromere regions showed the structural features of nucleosome-enriched sequence are very different from those of overall genomic sequence, suggesting these structural features involve in chromatin organization, acting as generator or repressor of nucleosome formation. Furthermore, differentially expressed genes exhibit different nucleosome occupancy patterns and chromatin structures in promoter regions. This observation indicated that these structural features play an important part in nucleosome organization and gene regulation, implying that the former may bridge the gap between nucleosome organization and gene expression.

Our findings illustrated the power of these structural features in predicting nucleosome occupancy and positioning. We used the least angle regression method to integrate all 12 structural features for predicting nucleosome occupancy. Besides those features such as the propeller twist and free energy which overlap with previous computational studies, we also find that the DNA denaturation, DNA-bending stiffness, Stacking energy and Z-DNA are effective in capturing nucleosome occupancy. These structural features capture more accurately in vivo nucleosome occupancy than sequence compositional features, consistent with a previous analysis which indicated that a major sequence signaling in vivo is a high-energy barrier rather than favorable sequence motifs [48]. Furthermore, we proposed a novel computational method, DLaNe, to detect peaks (valleys) of structural profiles to locate nucleosome positions. Most of these structural features have better performances than the existing methods in locating nucleosomes. We developed a meta DLaNe to integrate predictive power of six top-performing features. Based on the profiles of these structural features, we used a HMM model to locate nucleosomes. Our meta DLaNe method and the HMM model are more accurate than three recently proposed computational methods in locating nucleosomes, showing effectiveness of secondary structures in capturing nucleosome positioning signal. Our prediction method is a new addition to the arsenal of nucleosome positioning prediction.

## Methods

### Data used

We downloaded the experimental nucleosome occupancy data measured in recent studies [9,45,58]. In these works, based on the susceptibility of nucleosome-depleted sequences to MNase, MNase assay was used for the digestion of genomic sequences. Then, microarray [45,58] or massive parallel sequencing [9] techniques were adopted to determine nucleosome occupancy. The data of Lee et al. covered the whole *S. cerevisiae *genome at a higher resolution (4 bp) [45]. Kaplan et al. used parallel sequencing to determine genome-wide nucleosome occupancy [9]. The nucleosome intensity signals from these studies were represented as log ratio between nucleosomal DNA and genomic DNA, showing nucleosomes as peaks of about 150 bp long, surrounded by lower values corresponding to nucleosome-depleted regions. From these studies, the experimental nucleosome occupancy data were collected. We identified 1,000 well-positioned nucleosome and 1,000 nucleosomedepleted positions and extracted corresponding genomic sequences [58]. For genome-wide comparison of structural profiles and the patterns of nucleosome occupancy, we respectively used the experimentally derived in vitro nucleosome occupancy dataset from Kaplan's study [9] and in vivo data from Lee's study [45].

The complete *S. cerevisiae *genome (May 2006 build) and the genome annotation were downloaded from Saccharomyces Genome Database (SGD) [59]. To evaluate our prediction method, we compared it with three recent computational methods [8,14,54], whose predicted nucleosome positions were collected from their websites [8,14] or generated by the program [54]. All predictions were validated by the same reference dataset, a genome-wide atlas of nucleosome positions [45].

### Calculating structural profile

We analyzed a comprehensive list of structural features related to flexibility, curvature and energy of DNA sequences, including Aphilicity [36], B-DNA twist [30], Bendability [31], DNA-bending stiffness [33], DNA denaturation [35], Duplex free energy [39], Duplex disrupt energy [38], Propeller twist [29], Protein-DNA twist [32], Protein deformation [32], Stacking energy [34] and Z-DNA [37]. For each feature, a corresponding structural model has been constructed by specific experimental technique. A detailed discussion of these features can be found in [42,44].

We calculated the structural profiles of the above 12 features on *S. cerevisiae *genome. The calculation of a structural profile was divided into two steps. First, we converted each DNA sequence into a numerical sequence by replacing each dinucleotide or trinucleotide with a structural value. This transformation was based on experimentally determined structural models [44]. Second, we used a moving average to smooth the raw structural profiles, with a step of 10 bp and a window size of 100 bp. The final structural profile is a vector of values of the structural features, at a resolution of 10 bp, which can be adjusted as needed. We tried different window sizes ranging from 5 to 200 bp. The result showed that smaller window sizes (*< *75 bp) were not sufficient for value smoothing. On the contrary, bigger sizes (*> *150 bp) had too strong an averaging effect, smoothing out the differences among intrinsic structural patterns at different positions. Thus, to retain a sufficient smoothing effect and avoid much modification to the data, we used the window size of 100 bp rather than the nucleosome size (165 bp). Meanwhile, with the step size 10 bp for the sliding window, we obtained the structural values at a resolution of 10 bp. This smoothing constraint may slightly affect the results of following nucleosome locations. For example, if the predictive peaks of structural profiles locate within *± *35 bp around true nucleosomes, the predictions have a resolution of *± *40 bp.

### Locating nucleosomes through peak detection

**Figure 6:**
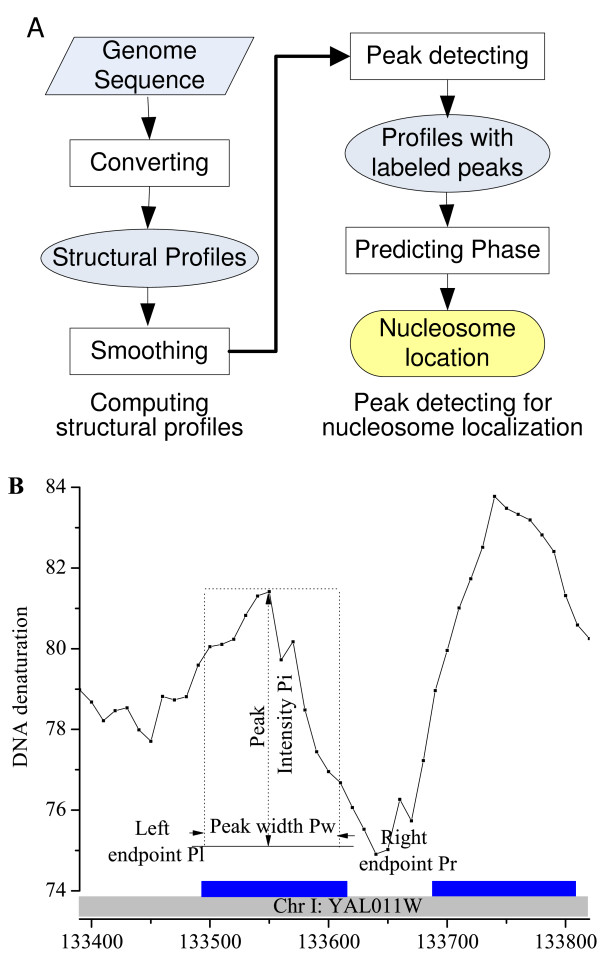
**Predicting nucleosome occupancy using DNA structural features**. (A) The flow chart of our new nucleosome position prediction approach DLaNe. (B) Illustration of parameters for peak detection.

According to our analysis, the structural profiles of nucleosome regions possess distinct characteristics, which are absent in the nucleosome-depleted sequences. For positively related features, the peak in the structural profile is a well-positioned property of the nucleosome. In contrast, the valley exists in nucleosome region for negatively related features. Thus, identification of nucleosome positions can be done by detecting peaks of structural profiles for positively correlated features, or detecting valleys for negatively correlated features. Since the detection of peaks and valleys is similar, we only present here peak detection procedure. As outlined in Figure 6(a), the approach is composed of two main parts, calculating a structural profile and detecting peaks to locate nucleosomes. The procedure for calculating structural profile was described in the previous section. In the following, we describe the peak detection model.

The structure vector *S *for a given sequence *s *can be obtained by the transformation procedure described above. The structural values, stored in *S*, can be plotted along the sequence, which may represent the changing patterns of the structural values, sketched in Figure 6(A). Meanwhile, we introduce four variables for defining a peak, i.e., peak intensity *P_i _*, left endpoint *P_l _*, right endpoint *P_r _*, and peak width *P_w _* (Figure 6(B)). To detect significant peaks, a predefined peak significance threshold *P_s _* needs to be determined empirically by an inspection of the average *P_i_*. In order to determine a *P_s _* for each chromosome, we tried different values in the range [0.1, 1]. The peak detection method performed best when *Ps *was chosen from [0.3, 0.6]. Then we can locate nucleosomes along the sequence as follows:

S.1) Filtering out noises of the structural profiles.

Although an initial smoothing is done to a structural profile, it may still have noises. Comparing with valid peaks, noises usually appear with low intensity and narrow shape. To filter out noises and meanwhile to minimize the amount of modification to the data, we adopt a median filtering to remove possible noises, i.e., for a position *p*, its value *S_p _* is replaced by the median value within a predefined window. Here the window size is the same as previous smoothing size (100 bp). Denote the median filter output of *S *as *SM*.

S.2) Determining the peak intensity threshold for each chromosome.

We then scan the noise-reduced structure vector *SM *with a sliding window. Since most common distances between adjacent nucleosome centers are approximately 165 bp (about 18 bp linker) in *S. cerevisiae *[19], the width of the window is set to 165 bp, other than the length of 147 bp as done in [45].

In order to determine the peak intensity threshold, the average peak intensity along the sequence is first calculated. Then relative to the average intensity, we define the peak intensity threshold to filter out the less intensive peaks. In each window, the difference between the maximal and minimal values is assigned to the peak intensity *P_i _* of the window. After scanning the whole sequence, we calculate the average *P_i _* of all windows, denoted as *AP_i_*. Based on *AP_i _* and a given peak significance threshold *P_s_*, the peak intensity threshold is determined as:

$$AP_{i}=EP_{i}=\sum_{i=1}^{\left\lfloor |SM|/P_{w}\right\rfloor }P_{i}/\left\lfloor |SM|/P_{w}\right\rfloor ,$$

where *SM *is the structure vector corresponding to sequence s, *Pw *is the predefined peak width, and

Ps∈(0,1].

Peak intensity threshold = *Ps*·*AP_i_*.

S.3) Searching for each peak's maximum position and endpoints.

To locate the concrete nucleosome position, we take the structural profiles and steric effect into account. The reason is that detailed nucleosome positions are influenced not only by high order chromatin structure, but also by repulsive and attractive interactions between neighboring nucleosomes. Steric exclusion prevents consecutive nucleosomes from overlapping in space, dictating relatively fixed lengths of linker DNA [60,61]. Thus, overlaps between two nucleosomes are not allowed owing to steric effect. A legal locating specifies positions for a set of non-overlapping 147-bp nucleosomes on *S. cerevisiae*. Thus, the detection of peaks follows the following rule. Given a peak intensity threshold, peak detection is performed by scanning the filtered structure vector *SM *. If the peak intensity of the window is less than the peak intensity threshold, viz., *P_i _< P_s_* ·*AP_i_*, there is no significant peak in this window, and the sliding window moves forward; otherwise, there exists a peak in the window. First, the position with the maximal *SM *value is regarded as *P_c_*. Since a well-positioned nucleosome is about 147 bp, *P_l _* and *P_r _* of this peak are correspondingly determined as follows, *P_l _* = *P_c _- *73, *P_r _* = *P_c _* + 73, where the value 73 is equal to half of the length of nucleosome. If there is more than one peak that exceeds the cutoff in the current window, the higher peak is chosen by selecting the maximal structural value in the window. Iteratively, the sliding window move forward to locate next nucleosome till it comes to the end of sequence. Then, each feature can be used to locate nucleosomes.

S.4) Integrating the predictions of individual features.

Furthermore, we introduce a Random Forest [62] based meta-predictor to integrate predictions of different structural features. Random Forest classifier is an ensemble classifier consisting of many decision trees with variations in structure and outputs the class voted by the majority individual trees [62]. First, the predictions of each feature are collected. For each prediction, the number of times that it is predicted by different features, the distance to its closest neighboring prediction and whether it is predicted by a certain feature are extracted as its features. Second, using the experimental nucleosome positions of one chromosome of yeast, we trained the Random Forest based meta predictor on the above selected features. Third, the trained meta predictor is applied to decide whether a prediction can be accepted. Finally, all accepted predictions are clustered if they are within 73 bp with each other, and the middle one in a cluster is taken as a meta prediction.

### Locating nucleosomes using a hidden Markov Model

The hidden Markov model (HMM) has been applied to infer nucleosome positions from genome-wide hybridization data [45,58]. As the profiles of these structural features are highly correlated with nucleosome occupancy, we also developed a HMM model to locate nucleosome from the structural profiles. Our implementation of HMM was based the HMM toolbox, which was downloaded from Murphy website http://www.cs.ubc.ca/~murphyk/Software/HMM/hmm.html. According to the resolution of these transformed structural profiles, the HMM model contained 16 distinct states, 15 nucleosome states and one linker state, which is different from previous models [45,58]. To apply the HMM model, the structural profiles of genomic sequences were first transformed as described above. After we obtained the structural profiles, we trained the model on Chromosome 3 based on Lee et al's reference nucleosome positions. Using the Viterbi algorithm, we applied the learned HMM model to compute the most-likely states. According to the predicted state sequence, we located the possible nucleosome positions.

The Additional file 2 provides the implementation of our DLaNe method based on structural features.

## Authors' contributions

YLG, JHG, SGZ and WZ conceived the study. YLG and WZ designed experiments. YLG performed the experiments. YLG and WZ analyzed the data and wrote the manuscript. All authors read and approved the manuscript.

## Supplementary Material

Additional file 1 **Supplemental Table 1**. Pairwise Pearson correlation coefficients among structural profiles of 12 different structural features across the whole *S.cerevisiae *genome. Supplemental Table S2. Pearson correlation coefficients between the 12 structural features and the G + C content across the whole *S.cerevisiae *genome. Supplemental Figure S1. The comparison between structural profiles and experimental nucleosome occupancy of *S.cerevisiae*. Here we show the other eight structural features of a 3 kb region around CHA1 promoter on chromosome 3, including three positive features (the first row) and five negative features (the second row). Supplemental Figure S2. The comparison between structural profiles and experimental nucleosome occupancy of *S.cerevisiae*. Here we show four typical related features of a 3 kb region around HIS3 promoter on chromosome 15, including DNA. Click here for file

Additional file 2 **The executable and source codes of DLaNe**. Click here for file
