# Supplementary material for: Structural features based genome-wide characterization and prediction of nucleosome organization
Source: BMC Bioinformatics. 2012 Mar 26;13:49. doi: 10.1186/1471-2105-13-49 (PMC3378464; doi:10.1186/1471-2105-13-49)
Supplement: Additional file 1 — Supplemental Table 1. Pairwise Pearson correlation coefficients among structural profiles of 12 different structural features across the whole S.cerevisiae genome. Supplemental Table S2. Pearson correlation coefficients between the 12 structural features and the G + C content across the whole S.cerevisiae genome. Supplemental Figure S1. The comparison between structural profiles and experimental nucleosome occupancy of S.cerevisiae. Here we show the other eight structural features of a 3 kb region around CHA1 promoter on chromosome 3, including three positive features (the first row) and five negative features (the second row). Supplemental Figure S2. The comparison between structural profiles and experimental nucleosome occupancy of S.cerevisiae. Here we show four typical related features of a 3 kb region around HIS3 promoter on chromosome 15, including DNA. [file 1471-2105-13-49-S1.PDF]

Supplemental Table 1

Pairwise Pearson correlation coefficients among structural profiles of 12 different structural features across the whole *S.cerevisiae* genome.

	DNA denaturation	Propeller twist	DNA-bending stiffness	Duplex disrupt energy	Bendability	Z-DNA	Stacking energy	Duplex free energy	Aphilicity	B-DNA twist	Protein-induced deformation	Protein-DNA twist
DNA denaturation	1											
Propeller twist	0.854	1										
DNA-bending stiffness	0.952	0.849	1									
Duplex disrupt energy	0.869	0.641	0.887	1								
Bendability	0.306	0.599	0.212	-0.023	1							
Z-DNA	-0.982	-0.878	-0.969	-0.859	-0.275	1						
Stacking energy	-0.959	-0.844	-0.903	-0.797	-0.372	0.956	1					
Duplex free energy	-0.976	-0.83	-0.972	-0.925	-0.213	0.981	0.957	1				
Aphilicity	-0.749	-0.814	-0.727	-0.534	-0.436	0.719	0.63	0.666	1			
B-DNA twist	-0.387	-0.121	-0.262	-0.268	0.07	0.279	0.198	0.249	0.599	1		
Protein-induced deformation	0.012	-0.122	0.021	0.071	-0.312	-0.045	0.012	-0.031	0.145	-0.033	1	
Protein-DNA twist	-0.358	-0.666	-0.416	-0.173	-0.627	0.336	0.258	0.288	0.767	0.199	0.307	1

Supplemental Table 2

The correlation coefficients between structural profiles of 12 different structural features and the G+C content across the whole *S.cerevisiae* genome, promoter and genic regions.

	The Whole Genome	Promoter	Genic region
Aphilicity	-0.755	-0.765	-0.719
B-DNA twist	-0.277	-0.287	-0.242
DNA-bending stiffness	0.796	0.806	0.761
Stacking energy	-0.808	-0.815	-0.771
DNA denaturation	0.822	0.834	0.78
Duplex free energy	-0.817	-0.825	-0.781
Duplex disrupt energy	0.812	0.821	0.779
Propeller twist	0.784	0.794	0.742
Protein deformation	0.022	0.031	-0.015
Protein-DNA twist	-0.529	-0.561	-0.484
Z-DNA	-0.773	-0.788	-0.697
Bendability	0.764	0.775	0.688

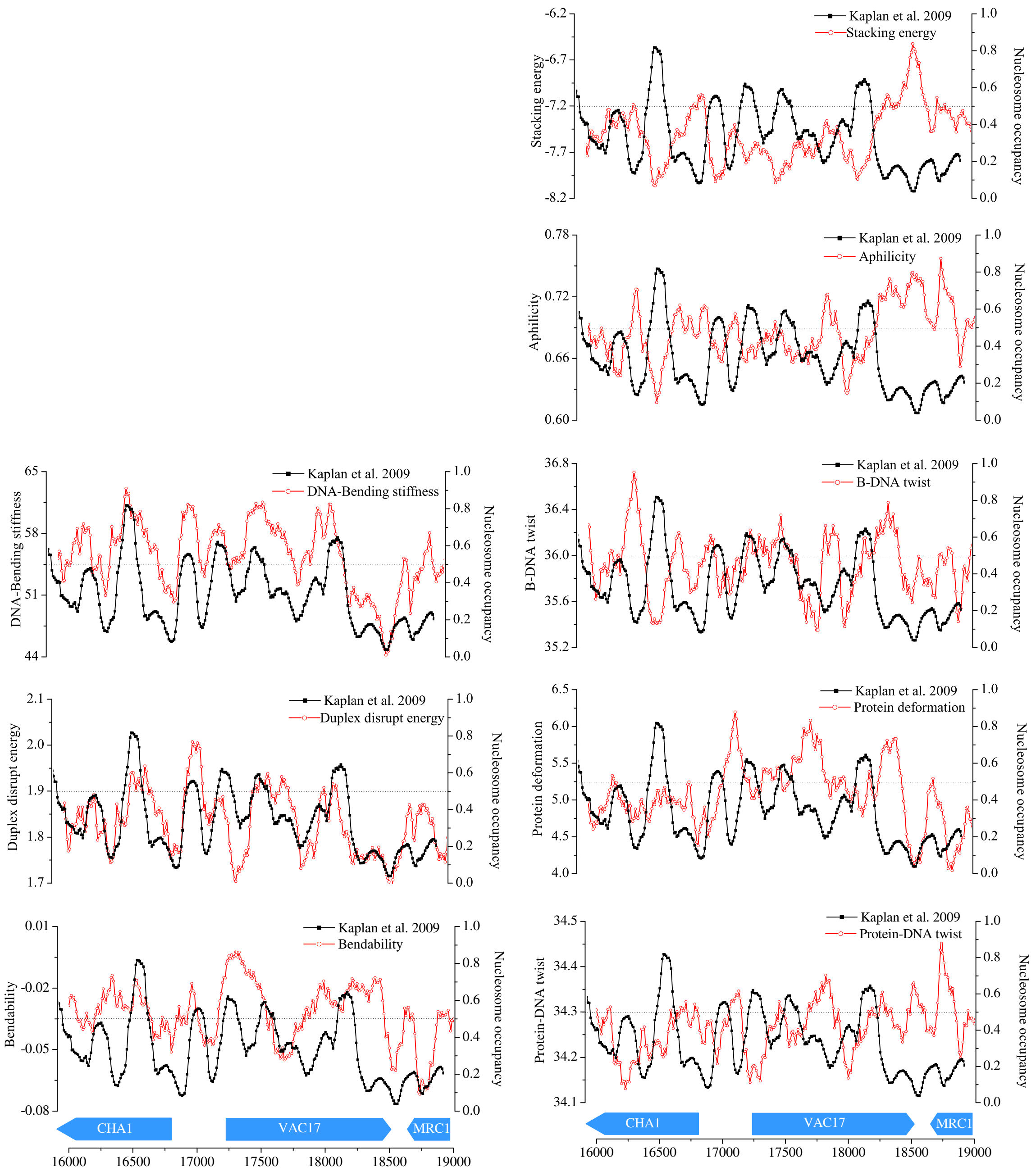

Supplemental Fig 1. The comparison between structural profiles and experimental nucleosome signal of *S.cerevisiae*. Here we show the other nine structural features of a 3kb region around CHA1 promoter on chromosome 3, including four positive features (the first row) and five negative features (the second row).

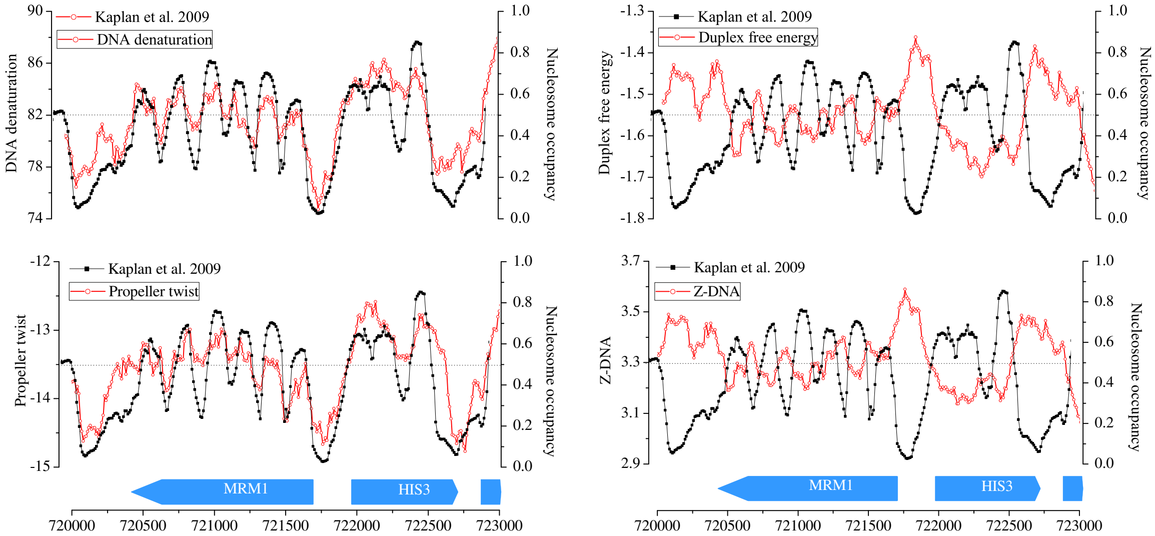

Supplemental Fig 2. The comparison between structural profiles and experimental nucleosome signal of *S.cerevisiae*. Here we show four typical related features of a 3kb region around HIS3 promoter on chromosome 15, including: DNA denaturation, Propeller twist, Duplex free energy, Z-DNA.
